# Modelling and Optimization of Machined Surface Topography in Ball-End Milling Process

**DOI:** 10.3390/ma17071533

**Published:** 2024-03-27

**Authors:** Renwei Wang, Bin Zhao, Dingzhong Tan, Wenjie Wan

**Affiliations:** 1 College of Mechanical and Electrical Engineering, Harbin Engineering University, Harbin 150001, China; 2 College of Power and Energy Engineering, Harbin Engineering University, Harbin 150001, China

**Keywords:** surface topography and roughness, ball-end milling, triangular approximation, Z-map model, optimal cutting parameters

## Abstract

In order to optimize machined surface topography, this paper presents a novel algorithm for simulating the surface topography and predicting the surface roughness of a ball-end milling process. First, a discrete workpiece model was developed using the Z-map method, and the swept surface of a cutter edge was represented using triangular approximation. The workpiece surface was updated (i.e., material removal process) using the intersection between the vertical reference line and the triangular facet under a cutting judgement. Second, the proposed algorithm was verified by comparing the simulated 3D surface topography as well as 2D surface profile and average roughness (*Sa*) with experimental measurements. Then, numerical simulation examples planed by the Box–Behnken design methods were carried out to investigate the *Sa* in the ball-end milling operation. The correlations of *S_a_* and cutting parameters were represented by a response surface reduced quadratic model based on the ANOVA results. Finally, the feed per tooth, radial depth of cut, and tilt and lead angles were optimized for improving the machining efficiency under the *Sa* constraints. This study presents an effective method for simulating surface topography and predicting the *Sa* to optimize the cutting parameters during ball-end milling process.

## 1. Introduction

Surface topography has a clear effect on the performance of components, such as their contact, friction, wear, and lubrication properties [1,2]. Surface topography is characterized by the surface roughness quantity for evaluating surface quality. Ball-end milling is one of the most widely used machining operations in various industries, such as aerospace, automobile, bioengineering and molding, as a semi-finish and finish method to replace grinding or polishing for improving the machining efficiency [3]. Therefore, the surface finish and dimensional accuracy of the final part are mainly determined by the ball-end milling operation in the finishing stage. According, the surface topography is easily influenced by many factors (cutting parameters) via ball-end milling. Chen et al. [4] investigated the effects of cutter inclination angle on the surface topography geometrical features using cutting experiments. Zhang et al. [5] investigated the ratio and product of the feed per tooth and radial depth of cut using an analytical model for reducing the surface roughness at a constant material removal rate. Peng et al. [6] investigated the effects of the cutter initial phase angle on the surface topography in micro-ball-end milling, and the surface texture was controlled by planning the noncutting tool path. Zhang et al. [7] used a modified cutting edge to develop a surface topography model considering the tool flank wear effect. Zhang et al. [8] considered stochastic tool wear for simulating the surface topography in micro-milling. Lotfi et al. [9] developed a modified cutter/workpiece engagement algorithm for simulating the surface model in five-axis ball-end milling considering the cutter deflection and cutter runout. Hao et al. [10] introduced a correction model of ball-end milling by wear and force deformation for simulating the surface topography. Omar et al. [11] proposed an integral model for simulating the surface topography and predicting the cutting force simultaneously, and investigated the effects of cutter axis tilt, cutter runout, tool deflection, systems dynamics, and face wear for the surface topography in the ball-end milling. Wang et al. [12] investigated the effects of cutting vibration on the surface topography in ball-end milling of thin-walled parts using a numerical method. Li et al. [13] investigate the geometrical errors imposed by the machine tool on the surface topography in five-axis ball-end milling processes, and evaluated quantity by means of modal coefficients. Biondani et al. [14] developed a surface topography model for investigating the effect of cutter edge micro-geometry on the surface topography and roughness based on an actual cutting-edge model and a judgment of minimum chip thickness. Therefore, numerical simulation has more applications compared with milling experiments for investigating the surface topography in ball-end milling operation. The surface topography simulation involves lower costs and is less time-consuming.

The surface topography simulation methods can be classed into three groups: solid modeling, iterative methods, and discretization. In the solid modeling methods [15], the material removal process is simulated by the Boolean operations between the cutter swept volume and workpiece. The iterative method was first proposed by Zhang et al. [16]. Mathematically, it is like solving the intersection of the swept surface of the cutter edge and the reference line at a selected workpiece point by solving the nonlinear equation using iterative methods. In the discretization methods, first, the cutter is dispersed into a series of units. The workpiece is constructed by means of the Z-map, N-buffer, Dexel and Voxel elements, cut plane, points set, etc. Then, machining time (or tool path) is dispersed, and at each discrete time point (or discrete cutter location point), the surface topography value (i.e., coordinate of workpiece surface) can be determined by the cutter edge unit intersecting with the workpiece. According to related research works, these are the main problems with the solid modeling as well as iterative methods and discretization. Indeed, in the solid modeling techniques, the computation of the intersection between geometrical objects is difficult and time-consuming. In iterative methods, a good initial value (also called starting point) must be determined first, while the determination of the initial value is not an easy task due to the nonlinearity of the equation of the swept surface and multiple intersections with the reference lines. In discretization methods, the computation efficiency and accuracy are low because of the plentiful discretization for the cutter and workpiece. However, the discretization method is still widely employed for modeling as a result of its extensibility and robustness with respect to solid modeling and iterative methods. Moreover, some study works have been performed to improve the accuracy and efficiency of the discretization method. Li et al. [17] introduced iterative methods to improve the discrete methods. In other words, they proposed a new initial value selection algorithm using discretization methods for the iterative methods. Xu et al. [18], Yang et al. [19], and Chen et al. [20] improved the discrete method using different interpolation methods. Therefore, the discretization method was adopted to develop the surface topography model. Meanwhile, an efficient algorithm for calculating the intersection point of the swept surface and workpiece was proposed, which is the key technology for simulating surface topography successfully.

The optimization of cutting parameters is simple and effective in machining process for obtaining the desired surface topography and roughness. Dikshit et al. [21,22] optimized the cutting parameters for minimizing the surface roughness using the response surface methodology in the ball-end milling of AL2014-T6. Zhou et al. [23] developed a modified grey relational analysis model for optimizing the surface roughness and residual stress in the ball-end milling of Inconel 718. Buj-Corral et al. [24] developed an artificial neural network for correlating surface roughness with cutting parameters to obtain the optimal machining strategy in the five-axis ball-end milling of W-Nr. 12344. Venkata-Rao et al. [25] considered the surface roughness as a constraint for reducing power consumption in the micro-ball-end milling of D2 steel. Sonawane et al. [26] used a response surface methodology for optimizing the cutting parameters and obtaining the minimum surface roughness in the high-speed ball-end milling of Inconel-718 thin cantilevers. Torres et al. [27] proposed a structured numerical simulation using several friction coefficient approximations, and adapted it to a pre-textured martensitic precipitation hardening stainless-steel. Cross et al. [28] investigated the surface topography of material measures using confocal microscope and atomic force microscope measurements, and it was found that the surface topography depends primarily on the spindle speed and much less on the feed per tooth, whereas differences between the milling tools are observed in the tendency to form substructures. Klauer et al. [29] found that the ploughing of the material instead of a cutting occurs when too-small tilt angles are applied, and investigated the tilt angle’s influence on the resulting roughness and machined geometry using micro-milling sinusoidal freeform surfaces at different tilt angles. Arruda et al. [30] optimized the surface roughness using the Taguchi method in the ball-end milling of AISI P20. Despite all this, there is a lack of research on improving the machining efficiency when surface roughness is applied as a constraint.

The objective of this study is to present a surface topography simulation algorithm for replacing a milling experiment to study the surface roughness during ball-end milling processes. As mentioned earlier, in order to model the surface topography, the intersection point between the swept surface of the cutter edge and the workpiece must be solved first. Hence, in the current study, an efficient numerical algorithm was proposed for calculating the intersection point between the swept surface and workpiece using an improved discretization method. To this end, first, the swept surface of the cutter edge was formulated by means of the homogeneous coordinate transformation and the kinematics of the ball-end milling cutter. Then, a discrete workpiece model was developed using the Z-map method, and the swept surface was represented using a triangular approximation. Finally, the intersection is calculated from the vertical reference lines and triangular facet. Once the intersection is solved, the mesh point of the workpiece surface can be updated by the intersection point with a smaller z-coordinate. The main contribution of the current study pertains to the central part of the proposed model, i.e., an algorithm for calculating the intersection point of the swept surface and the workpiece for updating the workpiece surface.

Henceforth, the paper is organized as follows. In Section 2, the surface topography simulation algorithm is presented in detail. In Section 3, milling experiments are carried out to valid the proposed algorithm. A response surface model has been developed for improving the machining efficiency under the average roughness (*Sa*) constraint. Finally, the conclusions of the work are summarized in Section 4.

## 2. Modeling of Machined Surface Topography

**Figure 1:**
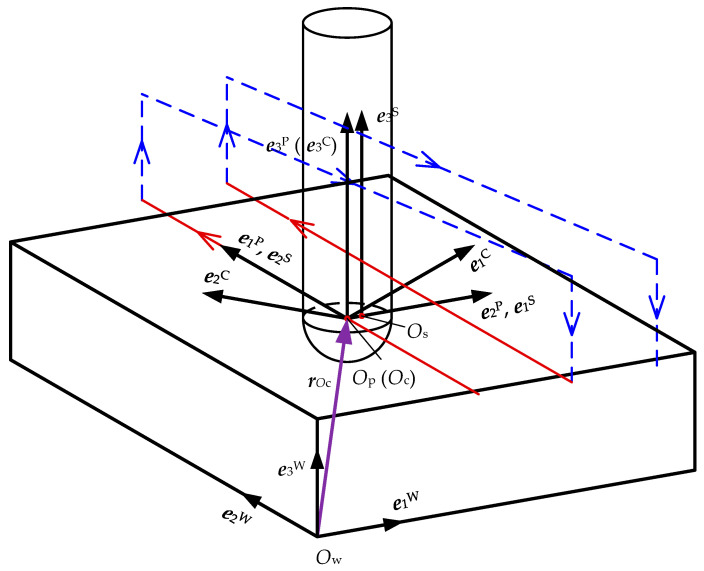
Definition of coordinate systems in ball-end milling of flat surface.

In order to obtain the equation of the swept surface of a cutter edge, the coordinate systems involved in the ball-end milling of flat surface processes are established as shown in Figure 1.

Machine tool coordinate system, *O*_m_ − *X*_m_*Y*_m_*Z*_m_ = {*O*_m_; ***e***_1_^M^, ***e***_2_^M^, ***e***_3_^M^}: the global coordinate system, *O*_m_, is placed at the origin of the machine tool, and ***e***_1_^M^, ***e***_2_^M^ and ***e***_3_^M^ can be determined by the kinematic structure of the machine tool.

Cutter coordinate system, *O*_c_ − *X*_c_*Y*_c_*Z*_c_ = {*O*_c_; ***e***_1_^C^, ***e***_2_^C^, ***e***_3_^C^}: the local coordinate system is fixed on the ball-end milling cutter. *O*_c_ is placed at the ball center and defined as the cutter location (CL) point. ***e***_3_^C^ is along the cutter axis. ***e***_1_^C^ is along the tangent of the cutter edge at the cutter tip. ***e***_2_^C^ is the cross product of ***e***_3_^C^ and the ***e***_1_^C^, ***e***_2_^C^ = ***e***_3_^C^ × ***e***_1_^C^. The cutter revolves around the spindle axis at an angular speed *ω*, and the cutter axis and the spindle axis show eccentricity, *e*.

Spindle coordinate system, *O*_s_ − *X*_s_*Y*_s_*Z*_s_ = {*O*_s_; ***e***_1_^S^, ***e***_2_^S^, ***e***_3_^S^}: local coordinate system attached to the spindle. *O*_s_ is placed at the intersection between the axis of the spindle and the *X*_c_*Y*_c_ plane. ***e***_3_^S^ is placed along the axis of the spindle, which is parallel to the ***e***_3_^C^. The spindle translates along the cutter path at ***r****_O_*_c_ *= (x_Oc_*, *y_Oc_*, *z_Oc_*). When the tilt and lead angles are equal to zero, *e*_1_^S^ is parallel to ***e***_1_^M^ and ***e***_2_^S^ is parallel to ***e***_2_^M^, while ***e***_3_^S^ is parallel to ***e***_3_^M^.

Process coordinate system, *O*_p_ − *X*_p_*Y*_p_*Z*_p_ = {*O*_P_; ***e***_1_^P^, ***e***_2_^P^, ***e***_3_^P^}: the local coordinate system is placed at the cutter path. *O*_p_ is placed at the cutter path and consistent with *O*_s_. ***e***_1_^P^ is along the feed direction and assumed to be ***e***_2_^W^, ***e***_3_^P^ is along the normal direction of the cutter center location surface and is parallel to ***e***_3_^M^, ***e***_2_^P^ is along the cross-feed direction and is also the cross product of ***e***_3_^P^ and ***e***_1_^P^, ***e***_2_^P^ = ***e***_3_^P^ × ***e***_1_^P^.

Workpiece coordinate system, *O*_w_ − *X*_w_*Y*_w_*Z*_w_ = {*O*_w_; ***e***_1_^W^, ***e***_2_^W^, ***e***_3_^W^}: the global coordinate system is attached to the workpiece, in which the cutter path and surface topography are described. *O*_w_ is placed at an arbitrarily selected point on the workpiece and the directions of ***e***_1_^W^, ***e***_2_^W^, and ***e***_3_^W^ are assigned to be consistent with the *O*_m_ − *X*_m_*Y*_m_*Z*_m_***e***_1_^M^, ***e***_2_^M^ and ***e***_3_^M^, respectively.

**Figure 2:**
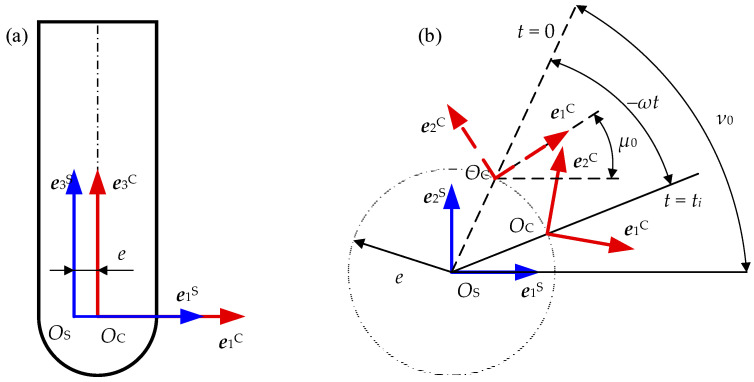
Definition of cutter runout: (**a**) front view; (**b**) top view.

The transformation of a selected point from *O*_c_ − *X*_c_*Y*_c_*Z*_c_ to *O*_s_ − *X*_s_*Y*_s_*Z*_s_ (see Figure 2) can be performed by a rotation around ***e***_3_^C^ and a translation along the *O*_s_*O*_c_. The transformation matrices ***M***^CS^ can be expressed as

(1) $$M^{\mathrm{CS}}=\left[\begin{array}{cccc} \cos\left(-\omega \cdot t+\mu_{0}\right) & -\sin\left(-\omega \cdot t+\mu_{0}\right) & 0 & e\cdot \cos\left(-\omega \cdot t+\upsilon_{0}\right)\\ \sin\left(-\omega \cdot t+\mu_{0}\right) & \cos\left(-\omega \cdot t+\mu_{0}\right) & 0 & e\cdot \sin\left(-\omega \cdot t+\upsilon_{0}\right)\\ 0 & 0 & 1 & 0\\ 0 & 0 & 0 & 1 \end{array}\right],$$

where *ω* = 2π*n*/60 (rad/s) is the angular speed of the cutter and *n* (r/min) is the rotational speed of the spindle, *t* is the machining time, eccentricity *e* is the distance from *O*_c_ and *O*_s_, *μ*_0_ is the initial angle of ***e***_1_^C^ and ***e***_1_^S^, and *υ*_0_ is the initial phase angle of the cutter runout.

**Figure 3:**
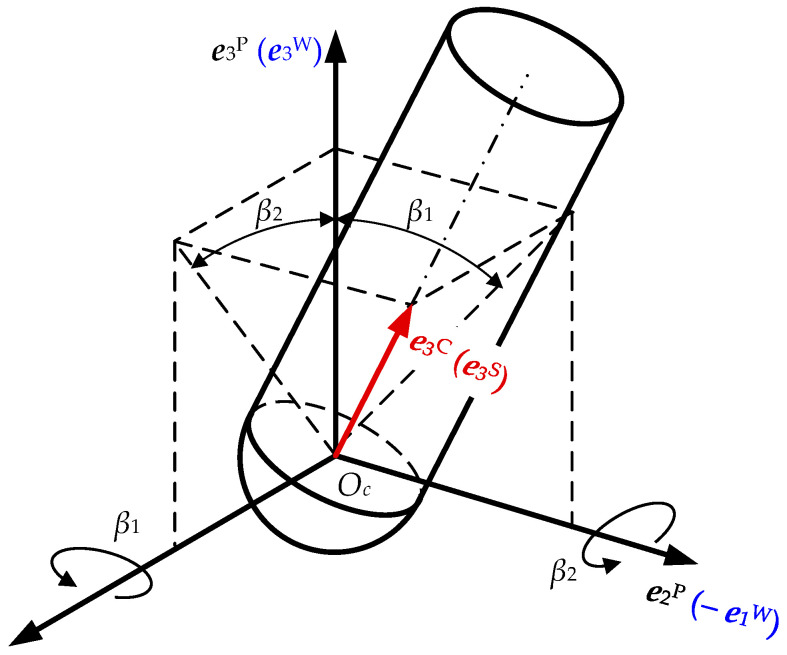
Definition of lead and tilt angles.

For a type-AB five-axis machining center, we must keep the cutter location point ***r****_O_*_c_ in the proper position and the cutter axis in proper orientation. The spindle translates along the cutter path, and rotates around ***e***_1_^M^ at *β*_2_, and rotates around ***e***_2_^M^ at *β*_1_ relative to the body of the machine tool (see Figure 3). Therefore, the transformation matrix ***M***^SW^ from *O*_s_ − *X*_s_*Y*_s_*Z*_s_ to *O*_w_ − *X*_w_*Y*_w_*Z*_w_ is defined as follows:

(2) $$M^{\mathrm{SW}}=\left[\begin{array}{cccc} \cos\beta_{1} & \sin\beta_{1}\cdot \sin\beta_{2} & -\sin\beta_{1}\cdot \cos\beta_{2} & x_{Oc}\\ 0 & \cos\beta_{2} & \sin\beta_{2} & y_{Oc}\\ \sin\beta_{1} & -\cos\beta_{1}\cdot \sin\beta_{2} & \cos\beta_{1}\cdot \cos\beta_{2} & z_{Oc}\\ 0 & 0 & 0 & 1 \end{array}\right],$$

where (*x_Oc_*, *y_Oc_*, *z_Oc_*) is the coordinate of in *O*_w_ − *X*_w_*Y*_w_*Z*_w_, *β*_1_ is the tilt angle, and *β*_2_ is the lead angle.

**Figure 4:**
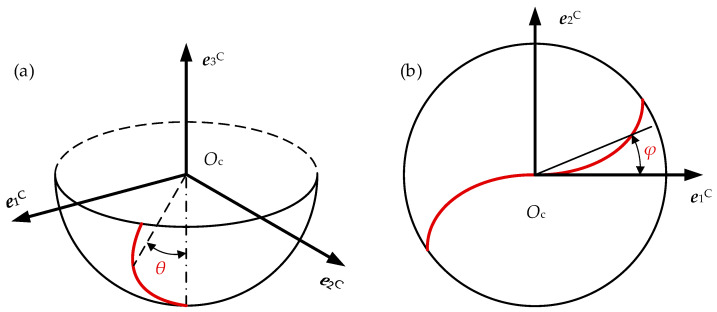
Definition of cutting edge model: (**a**) general view; (**b**) top view.

As shown in Figure 4, with the parameter of the axial position angle *θ* of a selected point p along the cutter edge, in a cutter coordinate system, *O*_c_ − *X*_c_*Y*_c_*Z*_c_, the spherical cutter edge can be expressed as:

(3) $$\begin{array}{cc} \left[\begin{array}{c} {x_{p}}^{C}\\ {y_{p}}^{C}\\ {z_{p}}^{C} \end{array}\right]=\left[\begin{array}{c} R_{0}\cdot \cos\theta \cdot \cos\varphi \\ R_{0}\cdot \cos\theta \cdot \sin\varphi \\ -R_{0}\cdot \cos\theta \end{array}\right], & \left(0\leq \theta \leq \tan\beta_{0}\right) \end{array},$$

where (*x*_p_^C^, *y*_p_^C^, *z*_p_^C^) is the coordinate of the point *P* in *O*_c_ − *X*_c_*Y*_c_*Z*_c_. *R*_0_ is the radius of the cutter. For a spherical cutter edge with equal lead, the lag angle *φ* can be calculated as follows:

(4) $$\varphi =\tan\beta_{0}\left(1-\cos\theta \right)+\left(j-1\right)2\pi /N_{c},$$

where *β*_0_ is the nominal helix angle as measured in the connection of the spherical and cylindrical cutter edges, *j* is the index of the cutter edge, and *N*_c_ is the number of cutter edges.

Given the cutter geometry as well as the cutter position and the cutter orientation, the swept surface of the cutter edge can be calculated in terms of homogeneous coordinate transformation. Mathematically, it is expressed as follows:

(5) $$\left[\begin{array}{c} {x_{p}}^{W}\\ {y_{p}}^{W}\\ {z_{p}}^{W}\\ 1 \end{array}\right]=M^{\mathrm{SW}}M^{\mathrm{CS}}\left[\begin{array}{c} {x_{p}}^{C}\\ {y_{p}}^{C}\\ {z_{p}}^{C}\\ 1 \end{array}\right],$$

where (*x*_p_^W^, *y*_p_^W^, *z*_p_^W^) is the coordinate of a selected point *P* on the swept surface in *O*_w_-*X*_w_*Y*_w_*Z*_w_.

**Figure 5:**
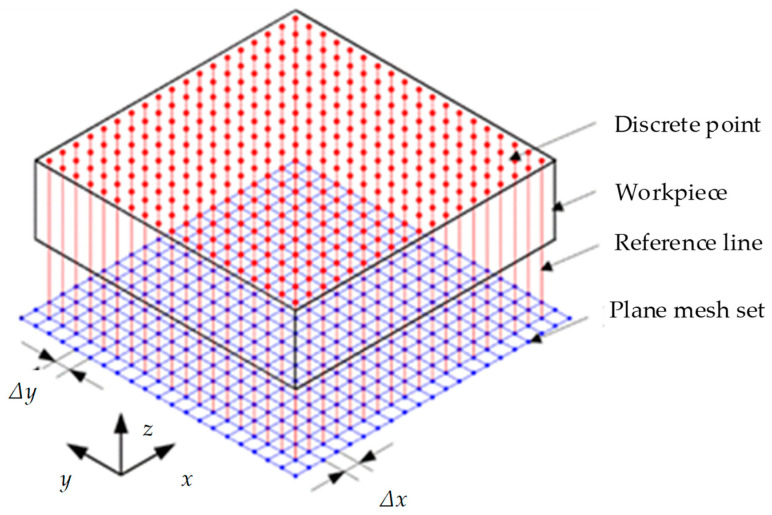
Z-map model of the workpiece.

**Figure 6:**
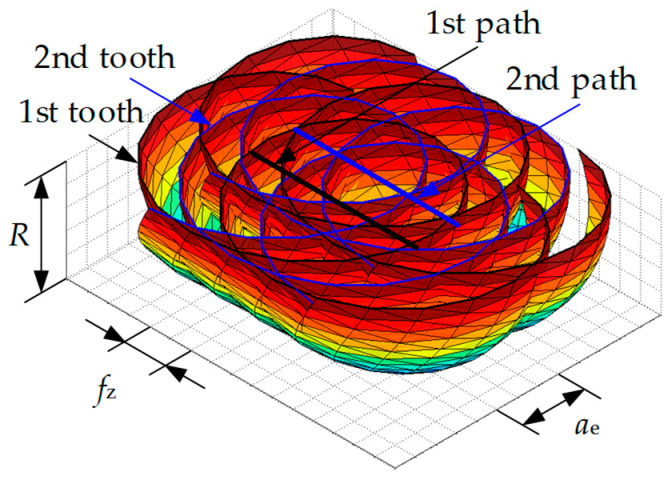
Triangular approximation of the swept surface.

**Figure 7:**
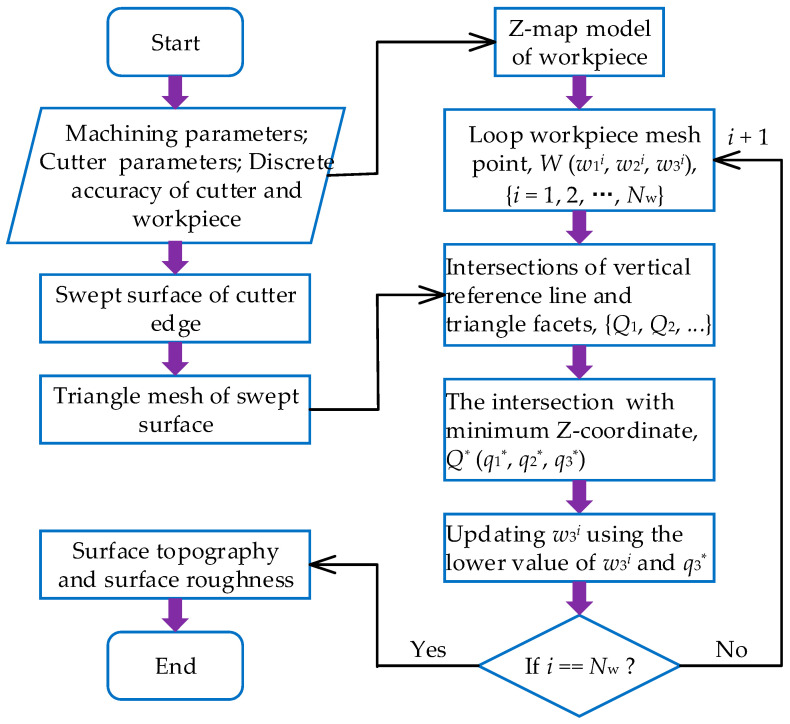
Sketched map of surface topography simulation algorithm.

The workpiece surface is updated (i.e., material removal process) based on a ray-triangle algorithm. First, the workpiece surface is dispersed into a series of discrete points using Z-map methods, as shown in Figure 5. Second, the swept surface of the cutter edge is dispersed into a series of triangle facets, as shown in Figure 6. Then, we update the discrete points of the workpiece surface using the intersection point between the vertical line of the workpiece surface and the triangular facet of the swept surface. Finally, we draw the surface topography as well as the surface profile, and calculate the surface roughness according to the coordinate of the discrete point of the workpiece surface. A sketched map of the surface topography simulation algorithm is shown in Figure 7.

## 3. Results and Discussion

### 3.1. Experimental Verification

In this study, the typical hot work die steel, AISI P20/3Cr2Mo (33 ± 3 HRC), combines a very good machinability with mirror polishing performance, and covers a wide variety of applications; it was chosen as the workpiece material here. The nominal chemical compositions and mechanical properties of the workpiece material are listed in Table 1 and Table 2, respectively. The workpiece samples were prepared as rectangular blocks (250 mm × 150 mm × 40 mm). Four samples were prepared. The top surfaces of each workpiece with sizes of 250 mm × 150 mm were divided into eight areas. Before milling experiments, the blocks were face-milled on the top and bottom surfaces to remove the heat treatment-related surface defects and flatness to eliminate errors in the experimental results. The cutter utilized in milling experiments was an integral carbide ball-end mill with two teeth (JH970100-Tribon, Seco Tools Company^®^, Troy, MI, USA) with a diameter of 10 mm and a 30° helix angle.

The flat surface milling tests were carried out on a five-axis computer numerical controlled vertical machining center (DMU 60P duo BLOCK, DMG^®^, DMP, Frankfurt, Germany) in a dry cutting environment with air-cooling. The milled samples were degreased by ultrasonic cleaning for 15 min in acetone and then rinsed with deionized water. The surface topography, as well as surface profile and *Sa*, of the machined surface was measured using an interferometer (Wyko NT9300, Bruker Corporation^®^, Billerica, MA, USA), and the vertical resolution was 0.1 nm. A rectangular region (1 mm × 1 mm) was chosen in order to compared and analyze the characteristics of surface topography during the ball-end milling process. In order to reduce accidental errors in the measurement, three different regions of the machined surface topography were measured under the same machining parameters. According to the engineering and cutting parameters manual, the value range of feed per tooth is 0~1 mm, the value range of the radial depth of cut is 0~1 mm, the value range of the axial depth of cut is 0~1 mm, the value of the spindle speed is 3000~10,000 r/min, and the lead and tilt angles are 0~45°. In order to deeply understand the influences of changes in cutting parameters on the five-axis ball-end milling process, five values of each factor have been selected according to the actual engineering and cutting parameters manual, as shown in [31]. Three arbitrary experiments under different cutting parameters (see Table 3) were selected to validate the developed surface topography model.

The discrete precision of the workpiece was set to 0.01 mm, which is the less than the minimum value of 0.1*f*_z_ and 0.1*a*_e_. The discrete precision of the swept surface is that the angle interval Δ*θ* = 0.2° and Δ*t* is the time it takes the cutter to rotate 2°.

**Figure 8:**
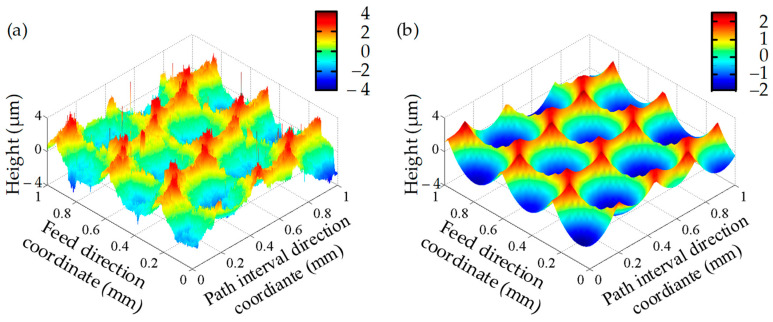
3D surface topography: (**a**) experimental; (**b**) simulated.

**Figure 9:**
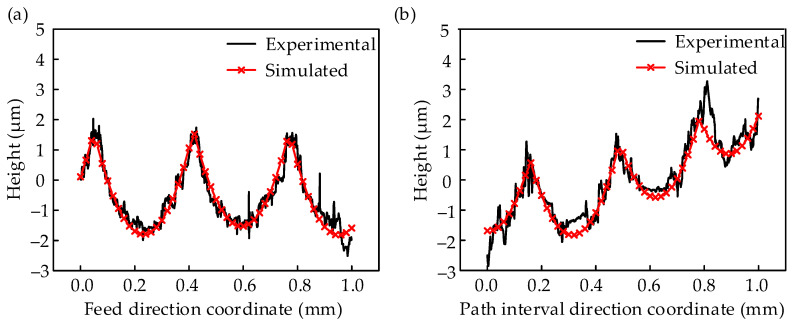
2D surface profile: (**a**) experimental; (**b**) simulated.

The predicted surface 3D topography and 2D surface profiles were compared with the experimentally measured results from the second trial, as shown in Figure 8 and Figure 9. The results show that the experiments and simulations were very similar to each other, both in the numbers of peaks and valleys and their distribution. There is a certain difference between the simulated and experimental results, such as the fact that the surface topography and profile were smooth in the simulations, while they were rough in the experiments. The main difference between the simulated and experimental results is in the sliding; the abrasion and elastic strain of the material sliding were not considered in the simulation.

According to the simulated and experimental surface topography results, the average roughness, *Sa*, has been calculated and used to evaluate the developed algorithm quantitatively. The *Sa* of trials 1#, 2# and 3# are listed in Table 4. The derivation between the simulated and experimental results with a maximum deviation is 13.48%, which shows that the developed model has a high prediction accuracy when predicting the surface roughness. The standard deviations are all less 0.1, which indicates that the measurement results are relatively concentrated, and the values of dispersion are small.

The verified results from Figure 8 and Figure 9 and Table 4 indicate that the surface topography simulation algorithm developed for the ball-end milling of AISI P20 is reliable. Therefore, this surface simulation algorithm can accurately simulate the surface topography and roughness, and thus replace milling experiments.

### 3.2. Cutting Parameters Optimization

The *Sa* is a basic requirement of the machining product’s quality, and the surface quality is classified into several grades using the value of *Sa*. Therefore, the *Sa* has been taken as a response parameter of the milled surface, which is calculated with the help of simulated surface topography. The feed per tooth (*f_z_*), radial depth of cut (*a_e_*), tilt (*β*_1_) and lead (*β*_2_) angle are selected as the numeric factors because they all have clear effects on the surface topography and can be changed by adjusting the feed rate, tool path and tool orientation. The ranges of the level of each numeric factor were set to feed per tooth—0.1~0.5 mm/tooth, radial depth of cut—0.1~0.5 mm, tilt angle—−8~8° and lead angle—−8~8° for the response analysis using the Box–Behnken design. The designs of the surface topography simulation examples and the simulated *Sa* values are presented in Table 5.

**Figure 10:**
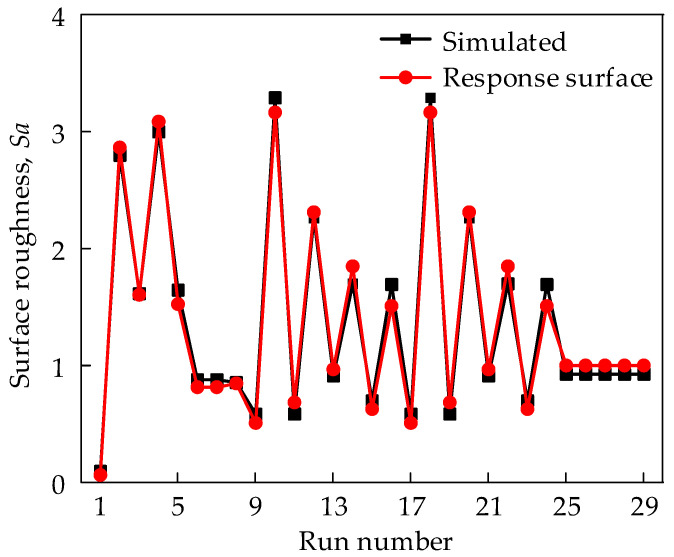
Comparison of average roughness between surface topography simulation and response surface models.

The results in Table 6 show that the model’s *F*-value of 145.73 implies the model is significant. There is only a 0.01% chance that a model *F*-value this large could occur due noise. Values of “Prob>*F*” less than 0.05 indicate that the model terms are significant. In this case, *f_z_*, *a_e_*, *β*_1_, *β*_2_, *f_z_* × *a_e_*, *f_z_* × *β*_1_, *f_z_* × *β*_2_, *f_z_*^2^ and *a_e_*^2^ are significant model terms. In contrast, values greater than 0.10 indicate the model terms are not significant; in this case, *a_e_* × *β*_1_, *a_e_* × *β*_2_, *β*_1_^2^ and *β*_2_^2^ are not significant model terms, and are reduced to simplify and improve the regression model, as shown in Equation (6). As shown in Figure 10, the comparison of predicted and simulated *Sa* indicates the high accuracy of the response surface model.

(6) $$\begin{array}{l} Sa=-0.2235+0.3955f_{z}+1.118a_{e}+0.0078\left(\beta_{1}+\beta_{2}\right)-8.2513f_{z}\cdot a_{e}\\ -0.3219f_{z}\left(\beta_{1}+\beta_{2}\right)+0.0116\beta_{1}\cdot \beta_{2}+16.681f_{z}^{2}+5.936a_{e}^{2} \end{array}$$

**Figure 11:**
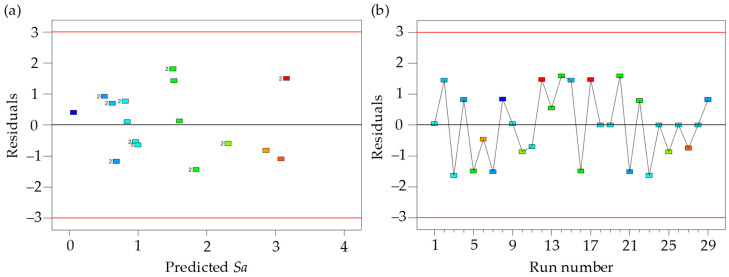
Residuals of response surface quadratic model: (**a**) residuals to predict surface roughness; (**b**) residuals to run number. The numbers represents the amount of similar results, and the colors make it easier to identify corresponding data points in (**a**,**b**).

**Figure 12:**
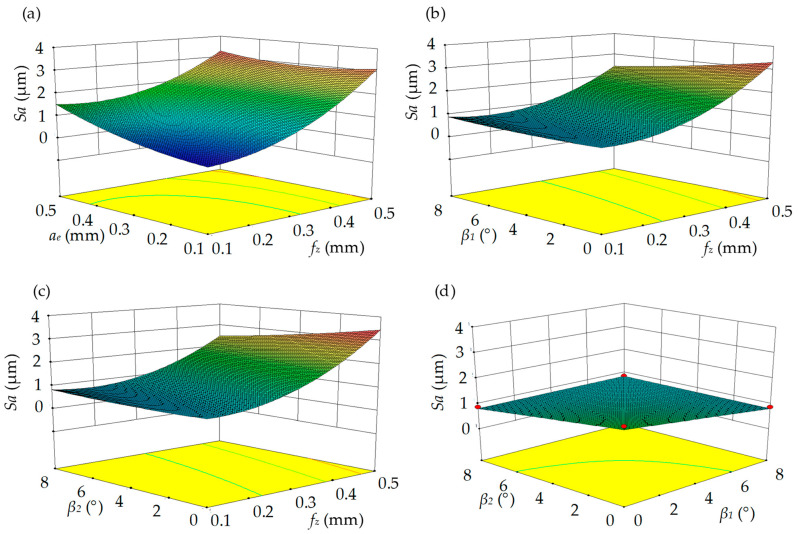
Interaction effect of machining parameters on average roughness: (**a**) feed per tooth and radial depth of cut; (**b**) feed per tooth and tilt angle; (**c**) feed per tooth and lead angle; (**d**) tilt and lead angles. Colors represent the design points above predicted value.

As shown in Figure 10, the predicted *Sa* using the response surface model was consistent with the simulated *Sa* using the proposed surface topography model, which indicates the high accuracy of the response surface model. The Studentized residuals are in the range of ±3, which is consistent with the results of the test. In addition, no abnormal data points were found in the residual plot (see Figure 11). The effects of machining parameters on the *Sa* are shown in Figure 12. The *Sa* increases with the increase in feed per tooth (*f_z_*) and radial depth of cut (*a_e_*) (see Figure 12a–c). The *Sa* decreases with the increase in tilt angle (*β*_1_) and lead angle (*β*_2_) (see Figure 12b–d). The feed per tooth (*f_z_*) and radial depth of cut (*a_e_*) have significant effects on material residual height, and the material residual height increases with the feed per tooth (*f_z_*) and radial depth of cut (*a_e_*). The tilt angle (*β*_1_) and lead angle (*β*_2_) can improve the interaction between the tool and workpiece, and reduce the *Sa*.

The response surface model’s accuracy has here been investigated. Next, the ability of the model to improve the machining efficiency by selecting the cutting parameters is analyzed (see Equation (7)). For this purpose, an optimal cutting parameter with the maximum material removal rate is obtained with an *Sa* of less than 0.7 by solving the response surface model; the optimal cutting parameters are *f_z_* = 0.2 mm/tooth, *a_e_* = 0.34 mm, *β*_1_ = 7.23° and *β*_2_ = 2.54°. The predicted *Sa* is 0.6841 μm, in contrast to the simulated *Sa*, which is 0.7791 μm. The devotion is −12.19%, which denotes the regression model is effective. In contrast to 20#, 21# and 23#, for which the *Sa* is less than 0.7 μm, the material removal rate can be improved from 0.03 to 0.068 at a growth of 126.67%. The result of the cutting parameters’ optimization is a substantial improvement in the machining efficiency under the surface roughness constraints.

(7) $$\left\{\begin{array}{ll} \max & f_{z}\times a_{e}\\ s.t. & Sa\leq 0.7,0<f_{z}\leq 0.5,0<a_{e}\leq 0.5,0\leq \beta_{1}\leq 8,0\leq \beta_{2}\leq 8 \end{array}\right.$$

## 4. Conclusions

In this paper, a novel surface topography simulation algorithm was developed for investigating the surface roughness in the five-axis ball-end milling process. The main conclusions as follows:

(1) A novel surface topography model was developed using triangular approximation and Z-map methods. The consistency between the simulated and experimental results shows that the model can replace the milling experiment when studying the surface topography and roughness during ball-end milling processes;
(2) A response surface-reduced quadratic model was developed based on the proposed surface topography simulation algorithm. The model can effectively characterize the correlation of *Sa* and cutting parameters (i.e., feed per tooth, radial depth of cut, tilt, and lead angles) based on ANOVA results;
(3) An optimization model was developed for improving the machining efficiency by means of the response surface model. The material removal rate (i.e., product of feed per tooth and radial depth of cut) can be improved effectively under the surface roughness constraints;
(4) The complex interaction between cutting edge and workpiece is neglected in the proposed model, so the cutting edge trajectory error, cutting edge micro-geometry and workpiece material deformation should be considered in the next study to secure a reliable prediction of surface topography.

## Figures and Tables

**Table 1:** Nominal chemical composition of AISI P20 steel (wt.%).

C	Si	Mn	Cr	Mo	Ni	Fe
0.28~0.40	0.20~0.80	0.60~1.00	1.40~2.00	0.30~0.55	0.05~0.10	Bal

**Table 2:** Mechanical properties of AISI P20 steel at room temperature.

Density (kg/m^3^)	Young’s Modulus (GPa)	Hardness (HRC)	Yield Strength (MPa)	Thermal Conductivity (W/m·K)
7800	207	30~36	1140	29.0

**Table 3:** Machining parameters of ball-end milling experiments.

No.	Spindle Speed, *n* (r/min)	Feed per Tooth, *f*_z_ (mm/tooth)	Radial Depth of Cut, *a*_e_ (mm)	Axial Depth of Cut, *a*_p_ (mm)	Tilt Angle, *β*_1_ (°)	Lead Angle, *β*_2_ (°)
1#	5000	0.36	0.25	0.3	−8	4
2#	6500	0.36	0.3	0.15	−12	0
3#	6500	0.56	0.2	0.1	−16	4

**Table 4:** Experimental and simulated roughness.

No.	Experimental *Sa* (μm)		Predicted *Sa* (μm)	Relative Deviation (%)
	Average Value	Standard Deviation		
1#	0.8158	0.0656	0.8227	0.85
2#	0.9289	0.0871	0.9087	−2.17
3#	1.8723	0.0985	2.1246	13.48

**Table 5:** Box–Behnken design and simulated results of average roughness.

Std	Feed per Tooth, *f_z_* (mm)	Radial Depth of Cut, *a_e_* (mm)	Tilt Angle, *β*_1_ (°)	Lead Angle, *β*_2_ (°)	Simulated Roughness, *Sa* (μm)
1	0.1	0.1	4	4	0.0937
2	0.5	0.1	4	4	2.7981
3	0.1	0.5	4	4	1.6139
4	0.5	0.5	4	4	2.9981
5	0.3	0.3	0	0	1.644
6	0.3	0.3	8	0	0.8779
7	0.3	0.3	0	8	0.8779
8	0.3	0.3	8	8	0.8536
9	0.1	0.3	4	0	0.5844
10	0.5	0.3	4	0	3.2895
11	0.1	0.3	4	8	0.5847
12	0.5	0.3	4	8	2.2598
13	0.3	0.1	0	4	0.9095
14	0.3	0.5	0	4	1.6984
15	0.3	0.1	8	4	0.6976
16	0.3	0.5	8	4	1.6937
17	0.1	0.3	0	4	0.5844
18	0.5	0.3	0	4	3.2895
19	0.1	0.3	8	4	0.5847
20	0.5	0.3	8	4	2.2598
21	0.3	0.1	4	0	0.9095
22	0.3	0.5	4	0	1.6984
23	0.3	0.1	4	8	0.6976
24	0.3	0.5	4	8	1.6937
25	0.3	0.3	4	4	0.925
26	0.3	0.3	4	4	0.925
27	0.3	0.3	4	4	0.925
28	0.3	0.3	4	4	0.925
29	0.3	0.3	4	4	0.925

**Table 6:** ANOVA for response surface quadratic model.

Source	Sum of Squares	df	Mean Square	*F* Value	*p*-Value (Prob>*F*)
Model	21.11	10	2.11	145.73	<0.0001
*f_z_*	13.76	1	13.76	949.88	<0.0001
*a_e_*	2.33	1	2.33	161.02	<0.0001
*β*_1_	0.35	1	0.35	23.86	0.0001
*β*_2_	0.35	1	0.35	23.86	0.0001
*f_z_* *× a_e_*	0.44	1	0.44	30.08	<0.0001
*f_z_ × β*_1_	0.27	1	0.27	18.31	0.0005
*f_z_ × β*_2_	0.27	1	0.27	18.31	0.0005
*a_e_ × β*_1_	0.011	1	0.011	0.79	0.3883
*a_e_ × β*_2_	0.011	1	0.011	0.79	0.3883
*β*_1_ × *β*_2_	0.14	1	0.14	9.50	0.0064
*f_z_* ^2^	3.07	1	3.07	212.15	<0.0001
*a_e_* ^2^	0.39	1	0.39	26.87	<0.0001
*β*_1_^2^	0.03	1	0.03	2.18	0.1615
*β*_2_^2^	0.03	1	0.03	2.18	0.1615
Residual	0.26	14	0.014		
Lack of fit	0.26	14	0.019		
Pure error	0.000	4	0.000		
Cor Total	21.37	28			

## Data Availability

Data are contained within the article.
